# Long Non-coding RNA HOTAIR Function as a Competing Endogenous RNA for miR-149-5p to Promote the Cell Growth, Migration, and Invasion in Non-small Cell Lung Cancer

**DOI:** 10.3389/fonc.2020.528520

**Published:** 2020-09-25

**Authors:** Hang Li, Zhigang Cui, Xiaoting Lv, Juan Li, Min Gao, Zitai Yang, Yanhong Bi, Ziwei Zhang, Shengli Wang, Sixuan Li, Baosen Zhou, Zhihua Yin

**Affiliations:** ^1^Department of Epidemiology, School of Public Health, China Medical University, Shenyang, China; ^2^Key Laboratory of Cancer Etiology and Intervention, University of Liaoning Province, Shenyang, China; ^3^Department of Clinical Epidemiology, Shengjing Hospital of China Medical University, Shenyang, China; ^4^School of Nursing, China Medical University, Shenyang, China; ^5^College of Medicine, The First Affiliated Hospital, Zhejiang University, Hangzhou, China

**Keywords:** non-small cell lung cancer, HOTAIR, miR-149-5p, HNRNPA1, ceRNA

## Abstract

Lung cancer is a leading cause of cancer death all around the world. Long non-coding RNAs (lncRNAs) have been confirmed to be involved in carcinogenesis of malignancies. However, the molecular mechanism of most lncRNAs in various kinds of cancers remains unclear. LncRNA HOTAIR and HNRNPA1 are reported to play an oncogenic role in non-small cell lung cancer, and the overexpression of HNRNPA1 is shown to promote the proliferation of lung adenocarcinoma cells. In our study, we find that the overexpression of HOTAIR could promote the proliferation and overexpression of miR-149-5p could inhibit the proliferation of lung cancer cells. Flow cytometric analysis determines that overexpression of miR-149-5p induces cell cycle arrest in the G0/G1 phases, whereas overexpression of HOTAIR decreases the proportion of G0/G1phase cells. Also, overexpression of HOTAIR promotes the migration and invasion ability of lung cancer cells, confirmed by the wound-healing and transwell assays, which are suppressed by overexpression of miR-149-5p. Furthermore, the dual-luciferase reporter assay indicates that miR-149-5p could bind both HOTAIR and the 3′UTR of HNRNPA1. In summary, we find that HOTAIR can regulate HNRNPA1 expression through a ceRNA mechanism by sequester miR-149-5p, which post-transcriptionally targets HNRNPA1, thus promoting lung cancer progression.

## Introduction

Lung cancer is the most lethal and frequently diagnosed malignant tumor. Approximately 2.09 million new cases were diagnosed, and 1.76 million died of lung cancer around the world in 2018 (1). In China, lung cancer remains the most common cancer with the highest incidence and mortality rate in 2015 (2). Given that the symptoms of lung cancer are not obvious in the early stage, the majority of lung cancer patients are diagnosed in the middle and late stages when they lose the best chance for treatment. Therefore, the overall 5-year survival rate of lung cancer is unfavorable (3). To protect the high-risk population and improve the prognosis of lung cancer, the development of effective screening biomarkers and potential therapy targets are important. Although considerable efforts are made to study the carcinogenesis of lung cancer, the underlying molecular mechanism is still unclear.

Tobacco smoking is an acknowledged environmental risk factor for lung cancer, nonetheless, it is estimated that 25% of lung cancer patients have no exposure to smoking, which suggests that genetically predisposed risk factors may play an important role in the carcinogenesis of lung cancer (4, 5). In recent years, a lot of evidence has shown that the non-coding RNAs such as microRNAs (miRNAs), and long non-coding RNAs (LncRNAs) play an important role in various kinds of cancers (6).

Long non-coding RNAs are a class of RNAs with no function of protein coding and are expressed uniquely in different tissues and cancers. Mounting evidence demonstrates that lncRNAs can exert on various cellular processes of carcinogenesis, such as cell differentiation, proliferation, apoptosis, and metastasis (6). LncRNAs function as an oncogene or tumor suppressor in various kinds of malignancies. Hox transcript antisense intergenic RNA (HOTAIR) is reported to play a role in carcinogenesis of various malignant tumors, including lung cancer (7–12).

Competing endogenous RNAs (ceRNAs) are transcripts that regulate each other at the post-transcriptional stage due to the shared miRNA response elements (MREs) (13). LncRNAs can function as ceRNAs to regulate the levels of miRNAs by competing for shared miRNAs binding to target genes, which results in the upregulation of the mRNA level of target genes. LncRNAs can play roles in regulating the expression level of target genes in a variety of malignant tumors, including lung cancer that has been reported in a large number of studies (14–20). Zhao et al. (21) find that lncRNA GMDS-AS1 could act as ceRNA to upregulate the mRNA of CYLD by sponging miR-96-5p. In addition, the intervention of the GMDS-AS1/miR-96-5p/CYLD axis could regulate the growth and apoptosis of lung adenocarcinoma cells (21). Yang et al. (18) find that LCAT1 function as a ceRNA for miR-4715-5p, which leads to the upregulation of the activity of the endogenous target of miR-4715-5p, Rac family small GTPase 1 (RAC1).

In the present study, we investigate the effect of miR-149-5p, HNRNPA1, and HOTAIR on lung cancer cells. We find that HOTAIR may act as a competing endogenous RNAs (ceRNAs) for miR-149-5p to upregulate the expression of HNRNPA1.

## Materials and Methods

### Cell Culture and Transfection

Human lung adenocarcinoma cell lines (A549, SPC-A-1) and normal lung bronchus epithelial cell line (HBE) were purchased from the Academy of Sciences of China (Shanghai, China). The cells were cultured in RPMI 1640 medium (Biological Industries, Israel) supplemented with 10% fetal bovine serum (Biological Industries, Israel) and 100 U/mL penicillin and 100 U/mL streptomycin (Biological Industries, Israel) and incubated in a 37°C cell incubator with a humidified atmosphere of 5% CO_2_. The cell transfection was performed with jetPRIME (Polyplus, France) for the dual luciferase reporter gene assay and INTERFERin (Polyplus, France) for siRNA-HOTAIR.

### Bioinformatics Analysis

LncRNA HOTAIR expression profiles of 91 lung adenocarcinoma and 65 normal lung tissues (GSE19188) were downloaded from Gene Expression Ominibus (GEO, https://www.ncbi.nlm.nih.gov/geo/). The data were analyzed with the GEO2R online tools developed by the National Center for Biotechnology Information (https://www.ncbi.nlm.nih.gov/geo/geo2r/, NCBI). The effect of expression of HOTAIR on prognosis of non-small cell lung cancer patients was analyzed by using online tools GEPIA (http://gepia.cancer-pku.cn/) (22). The MiRWalk2.0 (23) (http://zmf.umm.uni-heidelberg.de/apps/zmf/mirwalk2/), miRTarBase (http://mirtarbase.mbc.nctu.edu.tw/), and RNA22 V2 (https://cm.jefferson.edu/rna22/Interactive/RNA22Controller) were applied to predict the binding sites of microRNA on HOTAIR and mRNA of HNRNPA1.

### Quantitative Real-Time Polymerase Chain Reaction

The total RNA was isolated from cells by using RNAIso plus according to the protocol of the manufacturer (Takara, Japan). The concentration of the RNA samples was measured by using Nanodrop 2000 (Thermo Fisher Scientific, USA), and immediately, the RNA samples were reverse transcribed to cDNA utilizing the PrimeScript™ RT reagent Kit with gDNA Eraser (Takara, Japan). MicroRNAs were reverse-transcribed by using stem-loop primers, which were specifically designed and synthesized by Sangon (China). Quantitative real-time polymerase chain reaction (qRT-PCR) was performed by using 2 × SG Fast qPCR Master Mix (Sangon, China) on an Applied Biosystems 7500 Real-Time PCR System (Applied Biosystems, USA) according to the manufacturers' protocol. 2^−ΔΔ*CT*^ methods were performed to calculate the relative gene expression. The expression of lncRNA and mRNA was normalized to GAPDH, and the expression of microRNA was normalized to U6 small nuclear RNA. The sequences of the primers used in the present study are showed in Table 1.

**Table 1:** Primers sequences.

**Gene**	**Sequences (5^**′**^-3^**′**^)**
miR-149-5p forward	5′-CGUCUGGCUCCGUGUCUUC-3′
miR-149-5p reverse	5′-AGUGCAGGGUCCGAGGUAUU-3′
miR-149-5p RT primer	5′-GUCGUAUCCAGUGCAGGGUCCGA GGUAUUCGCACUGGAUACGACGGGAGU-3′
U6 forward	5′-AGAGAAGAUUAGCAUGGCCCCUG-3′
U6 reverse	5′-AGUGCAGGGUCCGAGGUAUU-3′
U6 RT primer	5′-GUCGUAUCCAGUGCAGGGUCCGA GGUAUUCGCACUGGAUACGACAAAAUA-3′
HOTAIR forward	5′-UCAGCACCCACCCAGGAAUC-3′
HOTAIR reverse	5′-AGAGUUGCUCUGUGCUGCCA-3′
GAPDH forward	5′-CAGGAGGCAUUGCUGAUGAU-3′
GAPDH reverse	5′-GAAGGCUGGGGCUCAUUU-3′

### Lentivirus Packaging and Transfection

The overexpression vector of HOTAIR and its control were named HOTAIR and HOTAIR-NC. The overexpression of miR-149-5p and its control were named miR-149 and miR-NC. The small interfering RNA (siRNA) for HOTAIR silencing and control were named siHOTAIR and siNC. Plasmid of HOTAIR overexpression, miR-149 mimics, and siHOTAIR were designed and constructed by GenePharma company (GenePharma, China). The lentiviral expression construct and the packaging plasmid were co-transfected to 293T to package the lentiviral particles. HOTAIR and miR-149-5p were packaged to lentivirus by the Genepharma company (GenePharma, China). We performed a preliminary experiment of lentivirus transfection to select the approximate transducing units of lentivirus for transfection in the next step, and 48 h after transfection, transfection efficiency was estimated by taking photos on the inverted fluorescence microscope. The fluorescence intensity of green fluorescent protein indicates the efficiency of transfection (Leica, Germany).

### Small Interfering RNA Synthesis for HOTAIR Knockdown and Transfection

To investigate the function of HOTAIR, three types of small interfering RNAs against HOTAIR (siHOTAIR) were synthesized by GenePharma Technologies (Shanghai, China). Transfection was performed with INTERFERin (Polyplus, France), and the efficiency of knockdown was examined by quantitative real-time PCR (qRT-PCR). The siHOTAIR with the highest knockdown efficiency was used for further study.

### Cell Proliferation Assay and Cell Confluence Determination

Cell proliferation was detected by using Celigo Imaging Cytometer (Nexcelom, USA). A549 and SPC-A-1 cells transfected with HOTAIR, miR-149, or siHOTAIR and their controls were counted by a Countstar IC1000 cell counter (Countstar, China) and seeded on a 6-well plate (Corning, USA) at a density of 5 × 10^4^ cells/well and incubated in the 37°C cell incubator with a humidified atmosphere of 5% CO_2_. After incubation for 24, 48, 72, and 96 h, cell confluence was measured by using a Celigo Imaging Cytometer (Nexcelom, USA).

### Transwell Assay

Upper chambers (Corning, USA) for transwell were placed in a 24-well plate, and A549 cells transfected with HOTAIR or HOTAIR-NC, SPC-A-1 cells transfected with siHOTAIR or siNC, and miR-149-5p or miR-NC were suspended in serum-free RPMI 1640 medium at a density of 2.5 × 10^5^ cells/ml. The upper chambers were seeded with cell suspensions (200 μl), and the bottom chambers were filled with 500 μl RPMI 1640 containing 10% FBS. After 36 h of incubating, cells migrated to the bottom chambers, and the chambers were washed three times with cold PBS buffer, then soaked in ice-bath methanol 15 min for fixing the cells. PBS buffer containing 1% crystal violet was used to stain the cells. The number of migrated cells were counted from five randomly selected fields under a light microscope.

### Wound-Healing Assay

Cell migration was also detected by wound-healing assay. Transfected A549 cells and SPC-A-1 cells were seeded in 12-well plates (Corning, USA), and artificial scratches were made by sterile pipette tips along the center of each well. When the cells reached over 90% confluence and the cell debris were removed by washing the cells three times with PBS buffer, photos were taken by using an inverted microscope (Nikon, Japan) in bright field instantly (0 h). After the cells were incubated in 37°C with serum-free RPMI 1640 for 24 h, photos were taken again using the identical method. The data was analyzed by using Image J 1.8.0 software (Bethesda, USA).

### Cell Cycle Analysis

Cell cycle analysis was performed by using flow cytometry. Transfected cells were harvested and washed twice with cold PBS buffer, and then 70% ethanol was added to fix the cells at 4°C overnight. The cells were stained with PI in the dark at room temperature for 30 min. The proportion of cells in each phase of the cell cycle was measured by Guava® easyCyte 12 (Millipore, USA) according to the manufacturer's protocol. The data was analyzed by ModFit LT 5.0 (Verity Software House).

### Western Blot

Cells were harvested and lysed by using the RIPA buffer containing protease inhibitors (Solarbio, China), and subsequently, the concentration of the protein samples were quantified by using the Enhanced BCA Protein Assay Kit (Beyotime, China). Protein samples were loaded and separated by 10% SDS-PAGE, and subsequently, they were transferred onto 0.45 μm PVDF membranes (Millipore, USA). The membranes were soaked in PBST buffer containing 5% skim milk at 37°C for 1 h, and then washed with PBST buffer thrice for 10 min. Subsequently, the membranes were incubated with the primary antibodies specific for HNRNPA1 (1:1,500, Proteintech, China) and β-actin (1:2,000, Bioss, China) at 4°C overnight, respectively. The membranes were washed three times with PBST buffer and were incubated with HRP-conjugated secondary antibody for 1 h at room temperature. Signals of the protein bands were detected by utilizing Immobilon ECL substrate (Millipore, Germany) and Azure Gel Imaging Systems C500 (Azure Biosystems, USA). Band intensity of western blot was measured by Image J 1.8.0 software (Bethesda, USA). The β-actin protein was selected as loading control.

### Cell Apoptosis Analysis

Cell apoptosis analysis was also performed utilizing flow cytometry. Transfected cells were harvested and washed twice with cold PBS buffer and were then stained with Annexin V-APC /7-AAD in the dark for 15 min. The cells were detected on Guava® easyCyte 12 (Millipore, USA) according to the manufacturer's protocol.

### Luciferase Reporter Assay

The luciferase reporter vectors were designed and manufactured by RioBio (Guangzhou, China). Cells (293 T) were seeded at 5 × 10^4^ cells/well in 24-well plates and were allowed to settle overnight. The next day, cells were co-transfected with pmir-h-HOTAIR-WT, pmir-h-HOTAIR-MUT, pmir-h-HNRNPA1-WT, or pmir-h-HNRNPA1-MUT reporter plasmids and mimics NC, miR-149-5p mimics accordingly; 24 h after transfection, cells were lysed using passive lysis buffer (Promega, USA), and the luciferase activity was measured by Synergy H1 Multi-Mode Reader (Biotek, USA) using the Dual-Glo Luciferase Assay System (Promega, USA) and normalized to renilla luciferase activity, respectively. Experiments were performed in triplicate.

### Statistical Analysis

Statistical analysis was performed using SPSS 21.0 software (SPSS Inc, USA) and GraphPad Prism 6.0 (GraphPad Software, USA). Two independent sample *t*-tests was performed to assess significant differences in measured variables among groups. All the experiments were performed in triplicate, and the data were presented as mean ± standard deviation (SD). A *P* < 0.05 was considered statistically significant.

## Results

### LncRNA HOTAIR Is Aberrantly Highly Expressed in Lung Adenocarcinoma

**Figure 1:**
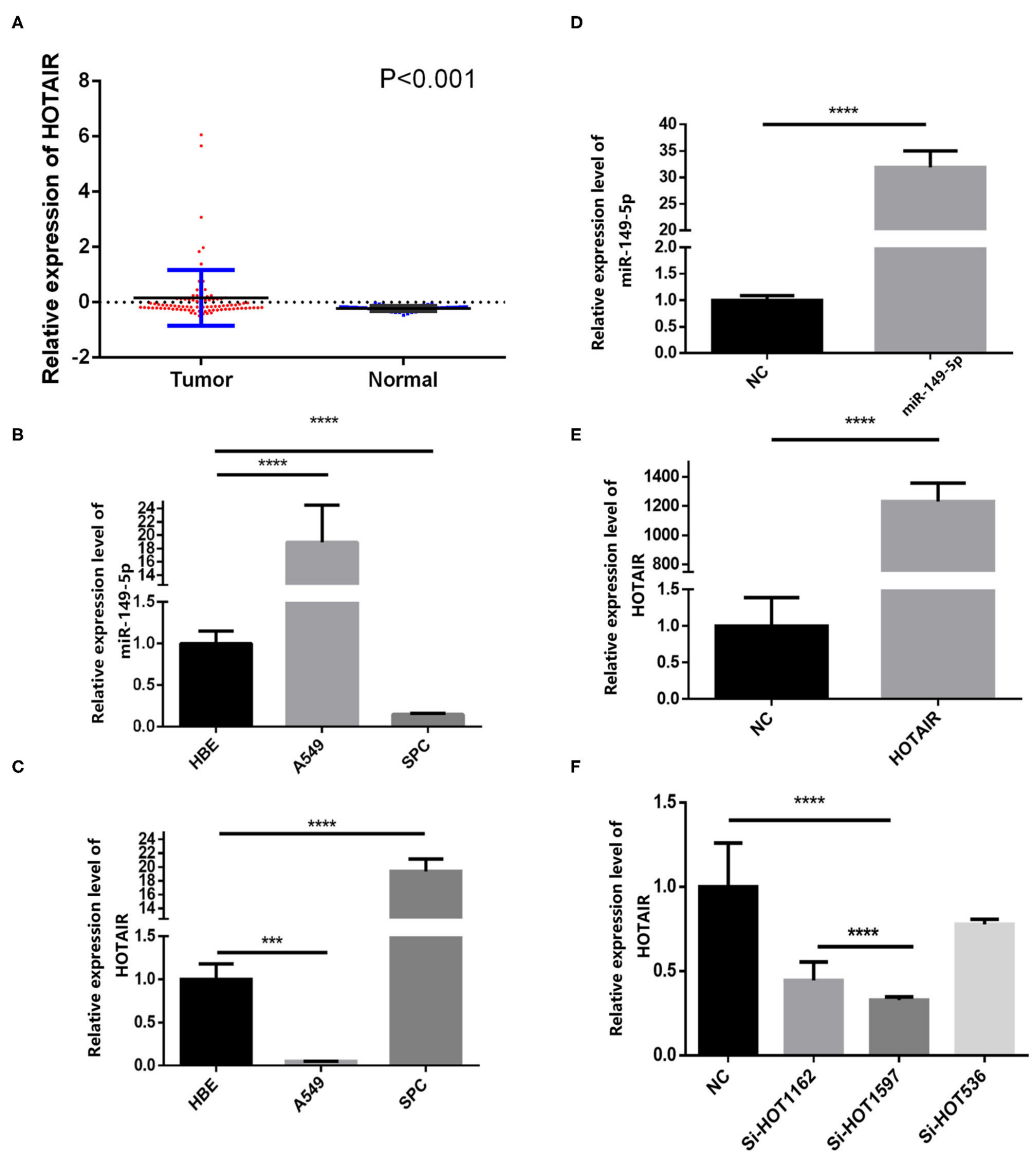
Relative expression of HOTAIR in non-small cell lung cancer tissues and normal lung tissues and QRT-PCR results of HOTAIR and miR-149-5p in A549 and SPCA1 cell lines and transfected cells. **(A)** Relative expression of HOTAIR in non-small cell lung cancer tissues and normal lung tissues from the GEO data set. **(B)** Background expression of miR-149-5p in HBE, A549, and SPC-A-1 cell lines. **(C)** Background expression of HOTAIR in HBE, A549, and SPC-A-1 cell lines. **(D)** Relative expression of miR-149-5p in SPC-A1 cells transfected by miR-149-5p overexpression lentivirus. **(E)** Relative expression of HOTAIR in A549 cells transfected by HOTAIR overexpression lentivirus. **(F)** Relative expression of HOTAIR in SPC-A-1 cells after transfection by small inferring RNAs (siHOT-1162, siHOT-1597, and siHOT-536). ***P < 0.01; ****P < 0.05.

We analyzed the gene expression data from GEO (GEO series accession No. GSE19188), and the results show that HOTAIR is aberrantly upregulated in 91 non-small cell lung cancer tissues compared with 65 adjacent normal lung tissues (Figure 1A).

### Aberrantly High Expression of LncRNA HOTAIR May Have an Unfavorable Prognosis

We analyzed the prognosis of lung cancer patients by using the GEPIA online tools, and the results of Kaplan-Meier analysis show that, compared with patients with high HOTAIR expression, disease-free survival (DFS) of patients with low HOTAIR expression was more favorable (HR = 1.4, *P* = 0.043; logrank *P* = 0.042) (Supplementary Figure 3).

### Background Expression of miR-149-5p and HOTAIR in Lung Cancer Cell Lines

The background expression level of miR-149-5p and HOTAIR in A549, SPC-A-1, and HBE cell lines were quantified by qRT-PCR (Figure 1B). The upregulated expression of HOTAIR was detected by using RT-qPCR in the SPC-A-1 cell line compared with A549 and HBE cell line (Figure 1C). The expression of miR-149-5p was lower in the SPC-A-1 cell line compared to that in A549 and HBE, and therefore, SPC-A-1 was chosen to perform miR-149-5p overexpressed lentivirus transfection, and the overexpression level of miR-149-5p was detected by qRT-PCR (Figure 1D). The background expression of HOTAIR in the A549 cell line was lower than that in the HBE cell line so that the A549 cell line was chosen to perform HOTAIR overexpressed lentivirus transfection (Figure 1E). Background expression of HOTAIR in the SPC-A-1 cell line was higher than that in HBE cell line; therefore, siHOTAIR and siNC were transfected to SPC-A-1 cell line to knock down HOTAIR expression to perform the gain- and loss-of-function experiments for investigating the effect of HOTAIR on lung cancer cells. The knockdown efficiency of HOTAIR was detected and quantified by qRT-PCR, and the result showed that siHOT-1597 had the highest knockdown efficiency (Figure 1F).

### miR-149-5p Inhibits the Proliferation of Lung Cancer Cells

**Figure 2:**
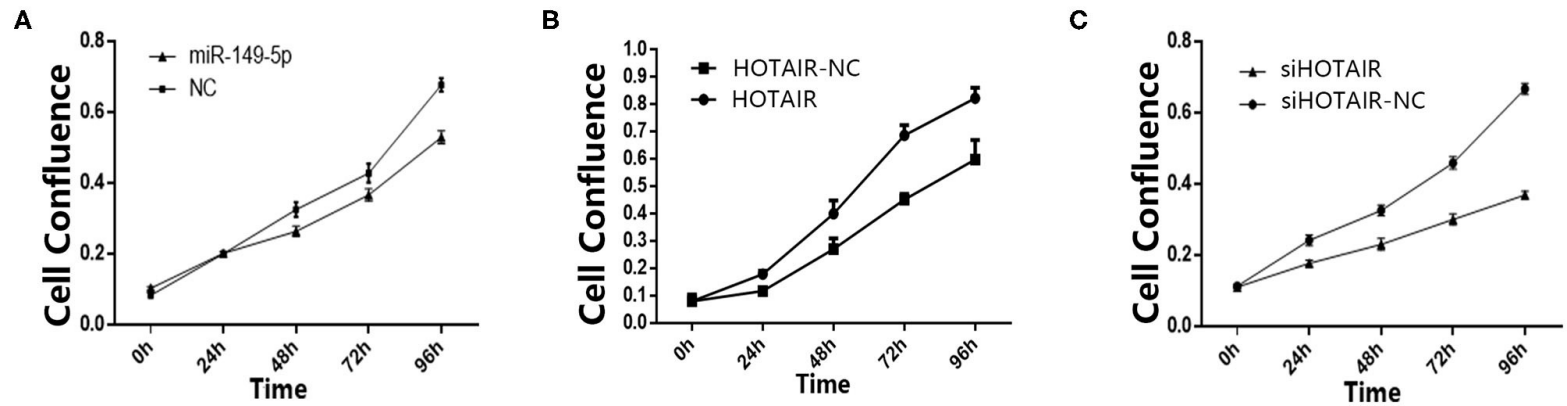
**(A)** Cell confluence of SPC-A-1 cells transfected by miR-149-5p overexpressed lentivirus after 24, 48, 72, and 96 h detected by Celligo. **(B)** Cell confluence of A549 cells transfected by HOTAIR overexpressed lentivirus after 24, 48, 72, and 96 h detected by Celligo. **(C)** Cell confluence of SPC-A-1 cells transfected by siHOT-1597 after 24, 48, 72, and 96 h detected by Celligo.

To investigate the effect of miR-149-5p on the proliferation of SPC-A-1 cells, transfected SPC-A-1 cells were planted on 6-well plates and photographed to measure the cell confluence by using Celigo Imaging Cytometer at 24, 48, 72, and 96 h after the cells were planted on 6-well plates. The cell growth activity of SPC-A-1 cells transfected with miR-149-5p overexpression lentivirus were suppressed compared with SPC-A-1 cells transfected with miR-NC (Figure 2A).

### LncRNA HOTAIR Promotes Cell Proliferation of Lung Cancer Cells

To elucidate the effect of HOTAIR on the proliferation of A549 cells, transfected A549 cells were planted on 6-well plates. After 24, 48, 72, and 96 h, the cells were photographed to measure the cell confluence by using a Celigo Imaging Cytometer. The cell proliferation was promoted in A549 cells transfected with HOTAIR overexpression lentivirus when compared to cells transfected with HOTAIR-NC (Figure 2B). Cell proliferation of SPC-A-1 cells transfected with siHOT-1597 was also detected, and results show that knockdown of HOTAIR could inhibit cell proliferation (Figure 2C).

### HOTAIR and miR-149-5p Play Roles in Cell Cycle of Lung Cancer Cell Line

**Figure 3:**
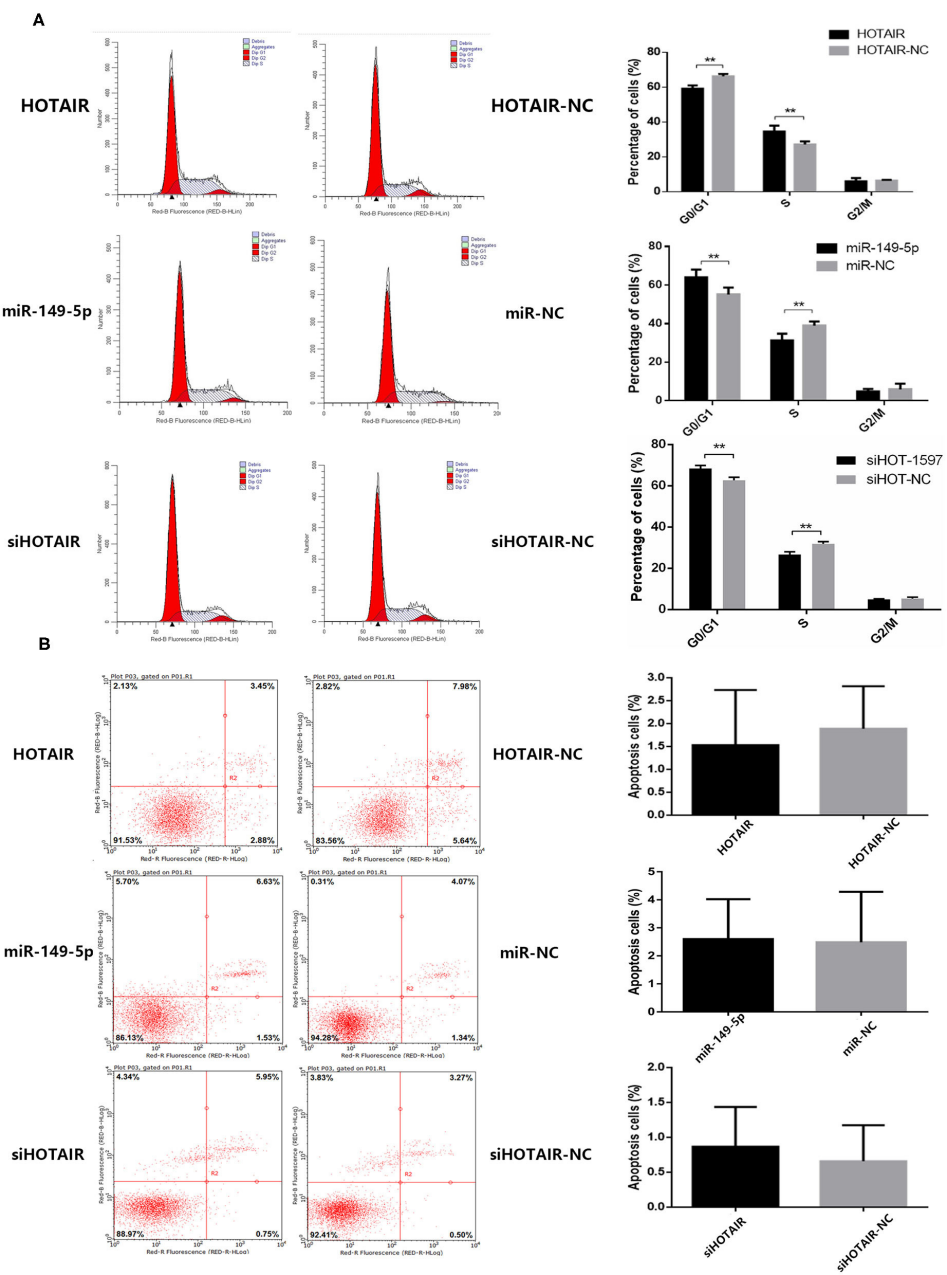
**(A)** Flow cytometry analysis of cell cycle distribution of lung cancer cells transfected with HOTAIR, miR-149-5p, and siHOTAIR. HOTAIR-transfected A549 cells show a decreased percentage of G0/G1-phase cells and increased S-phase cells (*p* < 0.05). miR-149-5p-transfected SPC-A-1 cells show an increased percentage of G0/G1-phase cells and decreased S-phase cells (*p* < 0.05). siHOTAIR-transfected SPC-A-1 cells show an increased percentage of G0/G1-phase cells and decreased S-phase cells (*p* < 0.05). **(B)** Flow cytometry analysis of apoptosis of lung cancer cells transfected with HOTAIR, miR-149-5p, and siHOTAIR. HOTAIR, miR-149-5p, or siHOTAIR have no effect on percentage of apoptosis apoptotic lung cancer cells (*p* > 0.05). **P < 0.05.

To investigate whether HOTAIR and miR-149-5p have an effect on the cell cycle of the lung cancer cell line, flow cytometry was performed. The percentage of cells in the G0/G1 phase of A549 cells transfected with HOTAIR was less than that of A549 cells transfected with HOTAIR-NC. The percentage of cells in the S phase of A549 transfected with HOTAIR was more than that of A549 transfected with HOTAIR-NC (Figure 3A). The percentage of cells in the G0/G1 phase of SPC-A-1 cells transfected with miR-149-5p was more than that of SPC-A-1 transfected with miR-NC. The S phase of SPC-A-1 transfected with miR-149-5p was less than that of SPC-A-1 transfected with miR-NC (Figure 3A). The percentage of cells in the G0/G1 phase of SPC-A-1 cells transfected with siHOTAIR-1597 was less than that of SPC-A-1 cells transfected with siNC. The percentage of cells in the S phase of SPC-A-1 transfected with siHOTAIR-1597 was less than that of SPC-A-1 transfected with siNC (Figure 3A). The results of cell cycle analysis suggest that HOTAIR may promote cell division and miR-149-5p may cause G0/G1 phase arrest.

### HOTAIR and miR-149-5p Have No Effect on Apoptosis of Lung Cancer Cell Line

To investigate whether HOTAIR and miR-149-5p have an effect on the apoptosis of lung cancer cell lines, flow cytometry was performed to estimate the percentage of apoptotic cells. A549 transfected with HOTAIR and HOTAIR-NC, SPC-A-1 cells transfected with miR-149-5p and miR-NC and cells transfected with siHOT-1597 and siNC were tested for apoptosis percentage. The results show that there is no statistically significant difference in the percentage of apoptotic cells between A549 cells transfected with the HOTAIR and HOTAIR-NC group (Figure 3B). No statistically significant difference was observed in the percentage of apoptotic cells between the SPC-A-1 cells transfected with miR-149-5p and cells transfected with miR-NC (Figure 3B). There was also no statistically significant difference in the percentage of apoptotic cells between the SPC-A-1 cells transfected with siHOTAIR and siNC (Figure 3B), indicating that HOTAIR or miR-149-5p may have no effect on the apoptosis of lung cancer cell lines.

### miR-149-5p Inhibited the Migration and Invasion Ability of the SPC-A-1 Cells

**Figure 4:**
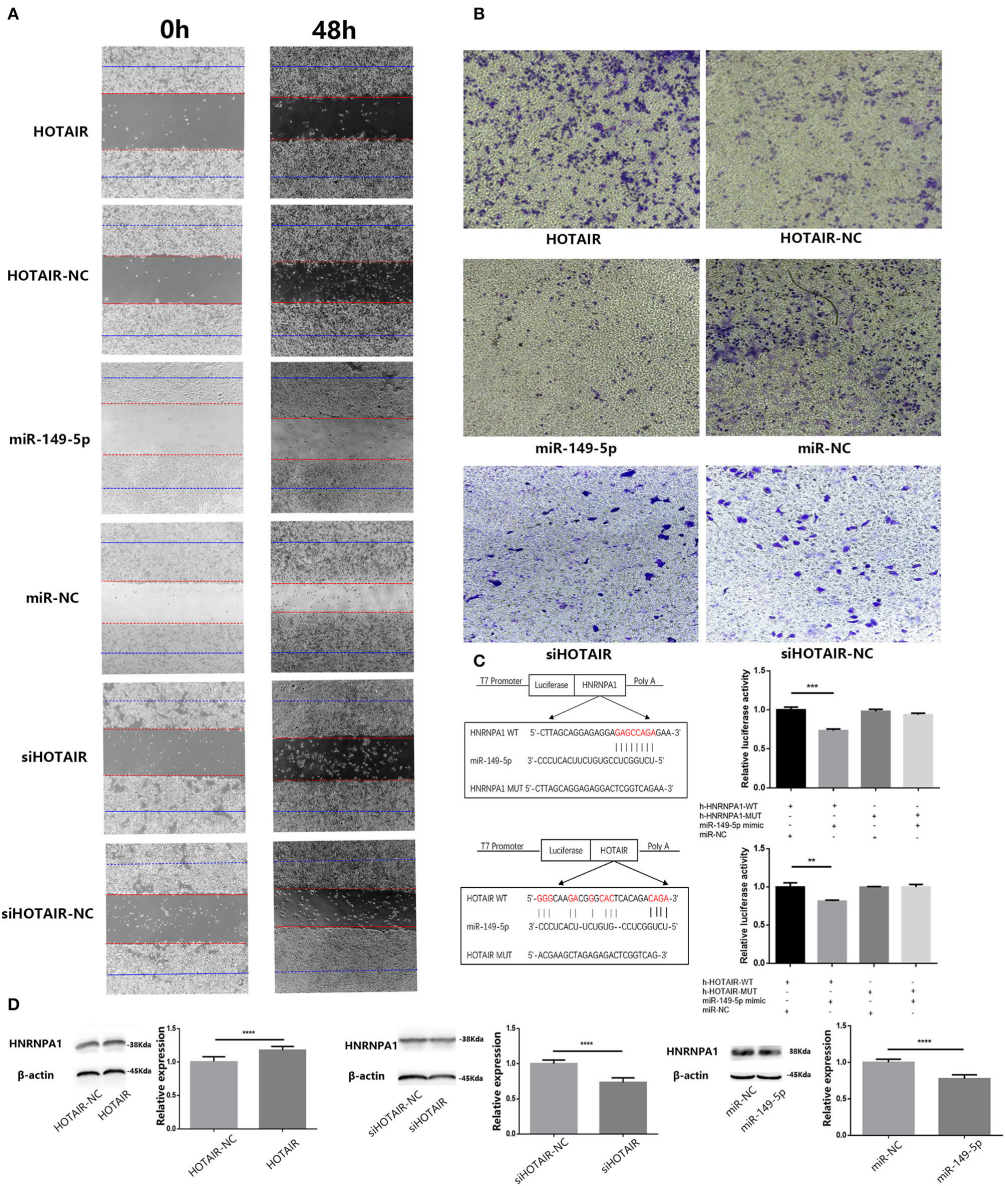
**(A)** Wound-healing assay was performed to analyze the migratory abilities of lung cancer cells (A549 and SPC-A-1) transfected with HOTAIR, miR-149-5p, and siHOTAIR for 0 and 48 h. Results show that HOTAIR can promote cell migration of A549 cells, miR-149-5p, can inhibit cell migration of SPC-A-1 cells and silencing HOTAIR can inhibit cell migration of SPC-A-1 cells. **(B)** Transwell assay was performed to analyze the invasion abilities of lung cancer cells (A549 and SPC-A-1) transfected with HOTAIR, miR-149-5p, and siHOTAIR. Results show that HOTAIR can promote cell invasion of A549 cells, miR-149-5p can inhibit cell invasion of SPC-A-1 cells, and silencing HOTAIR can inhibit cell invasion of SPC-A-1 cells. **(C)** Predicted binding site between HNRNPA1 and miR-149-5p and predicted binding site between HOTAIR and miR-149-5p. Luciferase activity in 293T cells of dual-luciferase reporter gene assay show that miR-149-5p can bind to HOTAIR and 3′UTR of HNRNPA1 (*p* < 0.05). **(D)** The protein level of HNRNPA1 in lung cancer cells (A549 and SPC-A-1) transfected with HOTAIR, miR-149-5p, and siHOTAIR detected by western blot analysis. **P < 0.01, ***P < 0.001, ****P < 0.05.

To investigate the potential effect of miR-149-5p on the migration and invasion ability of lung cancer cells, we performed wound-healing and transwell assays. The results of the wound-healing assay showed that the migration ability of SPC-A-1 cells transfected with miR-149-5p was inhibited compared with SPC-A-1 cells transfected with miR-NC (Figure 4A). Results of the transwell assay showed that invasion ability of SPC-A-1 cells transfected with miR-149-5p was inhibited compared with SPC-A-1 cells transfected with miR-NC (Figure 4B). The results suggest that miR-149-5p may inhibit the migration and invasion ability of SPC-A-1 cells.

### HOTAIR Can Promote the Migration and Invasion Ability of Lung Cancer Cells

To determine whether HOTAIR could affect the migration and invasion ability of A549, we performed wound-healing and transwell assays in transfected A549 cells. The results of the wound-healing assay show that the migration ability of A549 transfected with HOTAIR was promoted compared with group of A549 transfected with HOTAIR-NC (Figure 4A). The result of the transwell assay showed that the invasion ability of A549 transfected with HOTAIR was promoted compared with the group of A549 transfected with HOTAIR-NC (Figure 4B). The migration and invasion ability of SPC-A-1 cells transfected with siHOT-1597 was also detected by wound-healing and transwell assays, and results show that knockdown of HOTAIR could inhibit the migration and invasion ability of SPC-A-1 cells (Figures 4A,B).

### HOTAIR and HNRNPA1 Were Targeted by miR-149-5p

Results of bioinformatics analysis to predict miR-149-5p binding sites show the potential binding sites of miR-149-5p on HOTAIR and mRNA of HNRNPA1. Subsequently, we performed a dual-luciferase reporter assay in the 293T cell line to determine the direct binding between HOTAIR and miR-149-5p and the direct binding between HNRNPA1 and miR-149-5p. The results show that a significant reduction in luciferase reporter activity was observed in group of cells co-transfected with pmir-h-HOTAIR-WT and miR-149-5p mimics and group of cells co-transfected with pmir-h-HNRNPA1-WT and miR-149-5p mimics (Figure 4C).

### HNRNPA1 Protein Expression Is Promoted by HOTAIR and Inhibited by miR-149-5p

Results of the Western blot assay showed that HNRNPA1 protein expression is downregulated in SPC-A-1 cells transfected with miR-149-5p compared with the control group (Figure 4D). In A549 cells transfected with HOTAIR, HNRNPA1 protein expression is elevated compared with A549 cells that are transfected with HOTAIR-NC (Figure 4D). The HNRNPA1 protein expression is suppressed in SPC-A-1 cells transfected with siHOTAIR compared with SPC-A-1 cells transfected with siNC (Figure 4D). The results suggest that HNRNPA1 is elucidated to be targeted by miR-149-5p and HNRNPA1 expression is positively correlated with HOTAIR expression. The ceRNA axis of HOTAIR/miR-149-5p/HNRNPA1 may exist.

## Discussion

Cancer is considered to be a genetic disease traditionally, and recent research has demonstrated that epigenetic regulation, such as DNA methylation, histone deacetylation, chromatin remodeling, gene imprinting, and non-coding RNA (ncRNA) regulation, play indispensable roles in cancer development. Non-coding RNAs, such as microRNAs, long non-coding RNAs, and circRNAs, constitute more than 90% of the human transcripts. It has been demonstrated that non-coding RNAs exert an important functional role in carcinogenesis of malignant tumors (6, 24). In the present study, our results show that HOTAIR forms a competitive endogenous RNA mechanism with miR-149-5p and HNRNPA1. HOTAIR can inhibit the binding of miR-149-5p to HNRNPA1 mRNA by competitively sequestering miR-149-5p, and thereby decreasing the degradation of HNRNPA1 mRNA. Overexpression of HOTAIR could promote the migration and invasion of lung cancer cells and improve cell proliferation ability. Overexpression of miR-149-5p could inhibit the migration and invasion of lung cancer cells and cell proliferation. Overexpression of miR-149-5p arrests the cell cycle of lung cancer cells in the G0/G1 phase. The dual luciferase reporter gene assay showed that miR-149-5p binds to HOTAIR, and miR-149-5p also has a targeted binding relationship with HNRNPA1.

MicroRNA plays an important role in proto-oncogenes or tumor suppressor genes in malignant tumors and can regulate the occurrence and progression of malignant tumors. It has been demonstrated that miR-149 plays a role as a proto-oncogene or a tumor suppressor gene in a variety of malignant tumors (25–29). A study on colorectal cancer finds that the expression level of miR-149 in colorectal cancer tissues is significantly lower than that in adjacent normal tissues, and the expression level of miR-149 is inversely proportional to the expression level of FOXM1. Patients with low expression levels of miR-149 are more likely to present with a poor prognosis, such as lymph node metastasis, distant metastasis, or more malignant TNM grades, and miR-149 could bind to mRNA of FOXM1 and then suppress its expression, thereby inhibiting the proliferation, metastasis, and invasiveness of colorectal cancer cells, which suggests that miR-149 could play a role as a tumor suppressor gene in colorectal cancer (30). Aberrant expression of miR-149 is found in non-small cell lung cancer cells and is associated with invasive properties of lung cancer cells and increased epithelial-mesenchymal transition as reported by the study of Ke et al. (25) The researchers demonstrate that miR-149 could inhibit the expression of FOXM1 and reduce the FOXM1 protein level and inhibit the EMT process in non-small cell lung cancer by miR-149 overexpression and knockout of FOXM1 gene. HNRNPA1 is reported to be overexpressed in lung adenocarcinoma tissues and may play an oncogenic role in lung adenocarcinoma. In research by Liu et al. lentivirus-mediated RNA interference of HNRNPA1 was conducted in the A549 cell line, and expression of HNRNPA1 protein was successfully suppressed. They find that reduction of HNRNPA1 could inhibit cell proliferation of A549 cells partly by inducing cell cycle arrest in the G0/G1 phase and possibly acting by affecting the expression of telomerase and NF-κB activation. It is suggested that HNRNPA1 acted as an oncogene in lung adenocarcinoma (31). In our study, we find that HNRNPA1 is a downstream target gene of miR-149-5p, and miR-149-5p could inhibit the protein expression of HNRNPA1 in a lung cancer cell line. miR-149-5p could inhibit the proliferation of lung cancer cells, and high expression levels of miR-149-5p could lead to cell cycle arrest in the G0/G1 phase. Overexpression of miR-149-5p could inhibit the migration and invasion ability of lung cancer cells elucidated by the results of wound-healing and transwell assays. Results of the dual-luciferase reporter assay show that, miR-149-5p could bind to 3′UTR of mRNA of HNRNPA1, thus inhibiting the protein expression of HNRNPA1, which provided a biological plausible evidence for tumor suppressor role of miR-149-5p in lung cancer.

HOTAIR is a long-chain non-coding RNA of about 2.2 kb in length. Transcribed to the HOXC region, it could bind to the PRC2 and LSD1 complexes (32). The abnormally expressed HOTAIR is closely related to the occurrence of various malignant tumors, including breast cancer (33), colorectal cancer (34), lung cancer (35), and gastric cancer (36). It has been reported that HOTAIR indirectly regulates the expression of proto-oncogenes through acting as the miRNA sponge (8, 37). In a study on gastric cancer, HOTAIR could indirectly regulate the expression of HER2 by binding to miR-331-3p (8). A study of pancreatic cancer finds that HOTAIR could indirectly regulate NOTCH3 by binding to miR-613 (37). Our study elucidates the target binding of HOTAIR to miR-149-5p and the results of the dual luciferase reporter gene also validate the existence of competitive endogenous RNA mechanisms of miR-149-5p with HOTAIR and HNRNPA1.

## Conclusion

HOTAIR could promote the migration, invasion ability, and cell proliferation of lung cancer cells. miR-149-5p could inhibit the migration, invasion ability, and cell proliferation of lung cancer cells. HOTAIR may act as a competing endogenous RNAs (ceRNAs) for miR-149-5p to upregulate the expression of HNRNPA1.

## Data Availability Statement

The datasets generated for this study are available on request to the corresponding author.

## Author Contributions

HL performed the molecular biology studies and drafted the manuscript. ZYi participated in the design of the study. HL, ZC, ZYa, JL, XL, MG, ZZ, SW, YB, SL, and BZ performed the statistical analysis. All authors read and approved the final manuscript.

## Conflict of Interest

The authors declare that the research was conducted in the absence of any commercial or financial relationships that could be construed as a potential conflict of interest.
