# Supplementary material for: Long Non-coding RNA HOTAIR Function as a Competing Endogenous RNA for miR-149-5p to Promote the Cell Growth, Migration, and Invasion in Non-small Cell Lung Cancer
Source: Front Oncol. 2020 Sep 25;10:528520. doi: 10.3389/fonc.2020.528520 (PMC7545358; doi:10.3389/fonc.2020.528520)
Supplement: Supplementary file 1 [file Data_Sheet_1.PDF]

Supplementary materials

Jefferson
HOME OF SIDNEY KIMMEL MEDICAL COLLEGE

Computational Medicine Center

HOME ABOUT US OUR SCIENCE EVENTS LEARN! DATA/TOOLS/DOWNLOADS NEWS/INQUIRIES

RNA22 v2 results

Resulted have computed and are shown below. If there are no results shown, it means your chosen parameters yielded no results.
Note: The p-value represents the likelihood that the target site loci is random. That is, a lower p-value represents a greater chance that the loci contains a valid MRE

<table>
<tr><th>miR Name</th><th>transcript name</th><th>leftmost position of predicted target site</th><th>folding energy (in -Kcal/mol)</th><th>heteroduplex</th><th>p value</th></tr>
<tr><td>hsa-miR-149-5p MIMAT0000450</td><td>NR_047517.1 Homo sapiens HOX transcript antisense RNA (HOTAIR), transcript variant 1, long non-coding RNA</td><td>597</td><td>-16.40</td><td>ACGAAGCTAGAGAGAGAGCCAGA
        ||| | ||||||||
CCCTCACTTCTGTGCCTCGGTCT</td><td>2.34E-1</td></tr>
<tr><td>hsa-miR-149-5p MIMAT0000450</td><td>NR_047517.1 Homo sapiens HOX transcript antisense RNA (HOTAIR), transcript variant 1, long non-coding RNA</td><td>619</td><td>-21.00</td><td>AGGAGGGAAGAGA--GCGCCAGA
 |||| ||||| | | ||||||
CCCTCACTTCTGTGCCTCGGTCT</td><td>1.25E-1</td></tr>
<tr><td>hsa-miR-149-5p MIMAT0000450</td><td>NR_047517.1 Homo sapiens HOX transcript antisense RNA (HOTAIR), transcript variant 1, long non-coding RNA</td><td>716</td><td>-14.20</td><td>GGGCAAGACGGGCACTCACAGACAGA
||| || :|:||| || ||||
CCCTCACT-TCTGTG--CCTCGGTCT</td><td>8.86E-2</td></tr>
</table>

Figure S1 Putative binding sites of HOTAIR and miR-149-5p predicted by using RNA 22 (https://cm.jefferson.edu/rna22/Interactive/RNA22Controller)

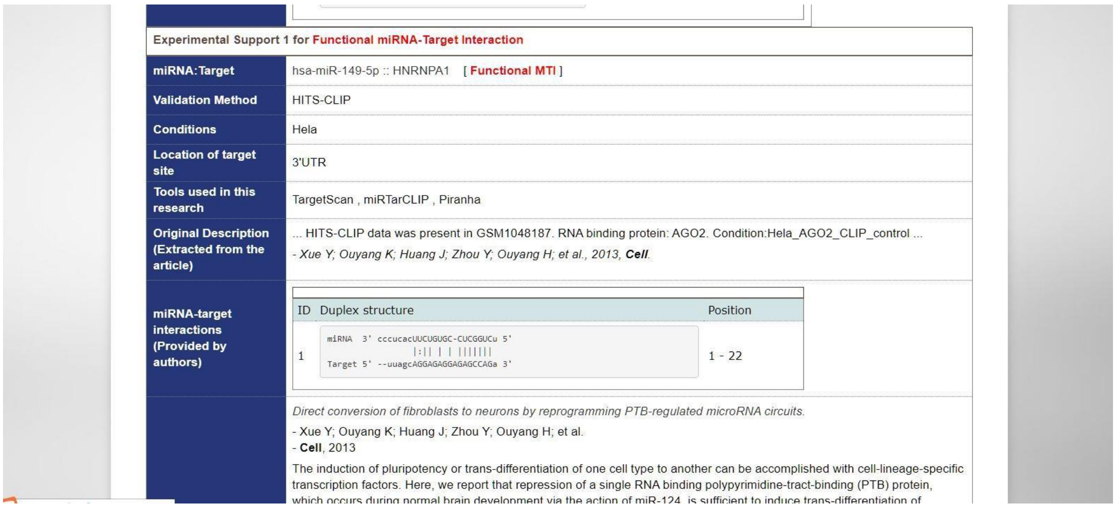

Experimental Support 1 for Functional miRNA-Target Interaction	
miRNA:Target	hsa-miR-149-5p :: HNRNPA1 [Functional MTI]
Validation Method	HITS-CLIP
Conditions	Hela
Location of target site	3'UTR
Tools used in this research	TargetScan , miRTarCLIP , Piranha
Original Description (Extracted from the article)	... HITS-CLIP data was present in GSM1048187. RNA binding protein: AGO2. Condition:Hela_AGO2_CLIP_control ...<br>- *Xue Y; Ouyang K; Huang J; Zhou Y; Ouyang H; et al., 2013,* ***Cell***.
miRNA-target interactions (Provided by authors)	ID: 1; Duplex structure: miRNA 3' cccucacUUCUGUGC-CUCGGUCu 5' / Target 5' --uuagcAGGAGAGGAGAGCCAGa 3'; Position: 1 - 22
	Direct conversion of fibroblasts to neurons by reprogramming PTB-regulated microRNA circuits.<br>- Xue Y; Ouyang K; Huang J; Zhou Y; Ouyang H; et al.<br>- **Cell**, 2013<br>The induction of pluripotency or trans-differentiation of one cell type to another can be accomplished with cell-lineage-specific transcription factors. Here, we report that repression of a single RNA binding polypyrimidine-tract-binding (PTB) protein, which occurs during normal brain development via the action of miR-124, is sufficient to induce trans-differentiation of

```
miRNA  3' cccucacUUCUGUGC-CUCGGUCu 5'
                 |:|| | | |||||||
Target 5' --uuagcAGGAGAGGAGAGCCAGa 3'
```

Figure S2 Putative binding sites of HOTAIR and miR-149-5p predicted by using miRTarBase (http://mirtarbase.mbc.nctu.edu.tw/)

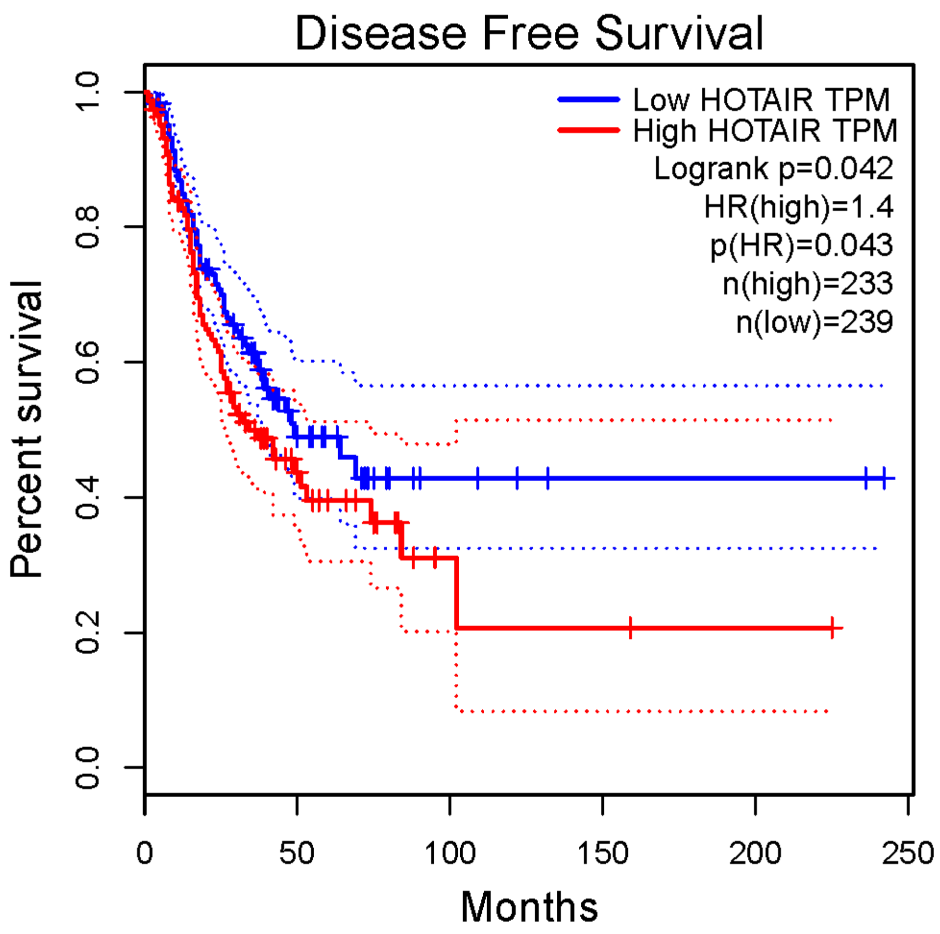

Figure S3 Disease free survival of patients with low expression and high expression of HOTAIR.

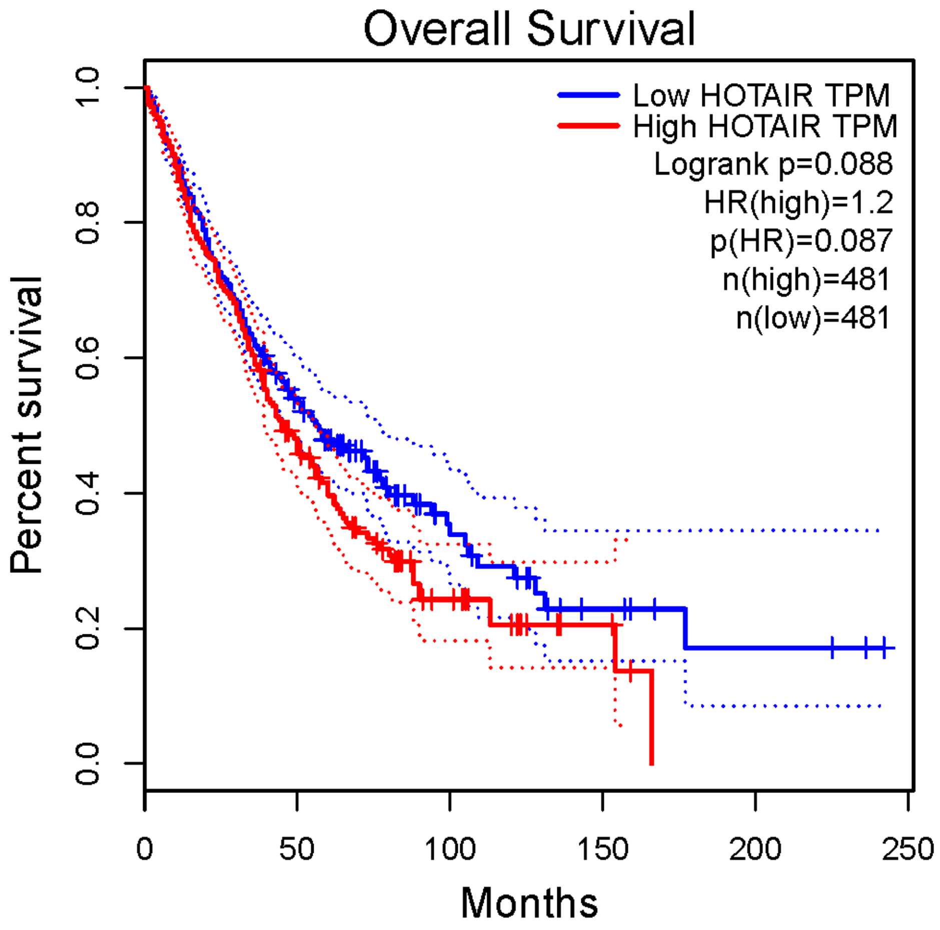

Figure S4 Overall survival of patients with low expression and high expression of HOTAIR.
